# Supplementary material for: Development of a computer-aided design software for the quantitative evaluation of aesthetic damage
Source: PLoS One. 2019 Dec 18;14(12):e0226322. doi: 10.1371/journal.pone.0226322 (PMC6919621; doi:10.1371/journal.pone.0226322)
Supplement: S1 Code site — 1. (ZIP) [file pone.0226322.s001.zip › [EESC_jr][Projeto]Relatorio_07_21/reports/templates_zip/backyard-high-converting-landing-page-template.zip!/backyard/backyard/contactthanks.html]

Thank You


## Thank You!

Good marketing makes the company look smart. Great marketing makes the customer feel smart, - Joe Chernov. Never doubt a small group of thoughtful, committed people can change the world. Indeed, it is the only thing that ever has, - Margaret Mead. The best way to predict the future is to create it, - Peter Drucker.

Learn More


Copyright Backyard 2015
